# Design and Synthesis of Novel 2D Porous Zinc Oxide-Nickel Oxide Composite Nanosheets for Detecting Ethanol Vapor

**DOI:** 10.3390/nano10101989

**Published:** 2020-10-09

**Authors:** Yuan-Chang Liang, Yen-Cheng Chang, Wei-Cheng Zhao

**Affiliations:** Department of Optoelectronics and Materials Technology, National Taiwan Ocean University, Keelung 20224, Taiwan; y41306@gmail.com (Y.-C.C.); qaz5263153@gmail.com (W.-C.Z.)

**Keywords:** 2D nanoarchitecture, hydrothermal, sputtering, composites, sensing response

## Abstract

The porous zinc oxide-nickel oxide (ZnO-NiO) composite nanosheets were synthesized via sputtering deposition of NiO thin film on the porous ZnO nanosheet templates. Various NiO film coverage sizes on porous ZnO nanosheet templates were achieved by changing NiO sputtering duration in this study. The microstructures of the porous ZnO-NiO composite nanosheets were investigated herein. The rugged surface feature of the porous ZnO-NiO composite nanosheets were formed and thicker NiO coverage layer narrowed the pore size on the ZnO nanosheet template. The gas sensors based on the porous ZnO-NiO composite nanosheets displayed higher sensing responses to ethanol vapor in comparison with the pristine ZnO template at the given target gas concentrations. Furthermore, the porous ZnO-NiO composite nanosheets with the suitable NiO coverage content demonstrated superior gas-sensing performance towards 50–750 ppm ethanol vapor. The observed ethanol vapor-sensing performance might be attributed to suitable ZnO/NiO heterojunction numbers and unique porous nanosheet structure with a high specific surface area, providing abundant active sites on the surface and numerous gas diffusion channels for the ethanol vapor molecules. This study demonstrated that coating of NiO on the porous ZnO nanosheet template with a suitable coverage size via sputtering deposition is a promising route to fabricate porous ZnO-NiO composite nanosheets with a high ethanol vapor sensing ability.

## 1. Introduction

Development of semiconductor oxide-based gas sensor has attracted much attention because of environmental problems associated with harmful air pollution. Binary metal oxides, such as SnO_2_, ZnO, WO_3_, NiO, Fe_2_O_3_, and Ag_2_O, are used for gas sensor materials [1,2,3,4]. Among various binary metal oxides, ZnO shows distinct gas-sensing properties towards various harmful gases. ZnO in a form of nanostructure is of potential interest for the gas-sensing material application because nanostructured ZnO has a high specific surface area that can improve its gas-sensing performance towards target gases [5,6,7]. Several variables, such as size and morphology, have been shown to markedly affect ZnO nanomaterial’s sensing performance. Among various morphologies of nanostructure for gas sensing applications, two-dimensional (2D) ZnO nanostructures have received high attention due to their special characteristics including nanometer scale in thickness and high specific surface area [8]. Besides, the sheet-like ZnO crystals are usually densely stacked by each other; the hierarchical structure may provide high spaces for target gases to diffuse into the inner region of the sensing material which improves their gas detection ability. For example, Mohammad et al. prepared hierarchical ZnO nanosheets which show high specific surface area and an increased proportion of exposed active planes. Those ZnO nanosheets display enhanced sensing responses to acetone vapor [9]. Ganesh et al. fabricated three-dimensional flower-like ZnO architectures which reveal fast response and recovery speeds to ammonia gas [10]. However, it is not enough for the ZnO nanosheets with a thin solid plate structure in gas-sensing property enhancement. Porous structure may further enhance gas-sensing sensitivity of sheet-like nanomaterials because porous structures usually have a large specific surface area, which act as abundant active sites for the reaction during gas-sensing tests [11]. Several reports on sheet-like ZnO nanomaterials have been proved that fabrication of porous surfaces on ZnO nanosheets is an efficient route to improve their gas detection ability towards various target gases [12,13].

Recently, construction of heterogeneously structured ZnO with a p-type sensing material has been shown an effective route to enhance its gas-sensing ability [14]. Cosputtering deposited ZnO-NiO p-n composite thin films with an optimal Ni content demonstrates an enhanced sensing performance to ethanol vapor [2]. P-type Co_3_O_4_-decorated ZnO nanowires synthesized by a thermal evaporation method show superior ethanol and NO_2_ gas sensing performances than that of the pristine ZnO nanowires [15]. These examples visibly demonstrate that the p-n heterojunction is beneficial to improve gas-sensing ability of the p-n composite structure. Among various p-type oxides, NiO has superior chemical and thermal stabilities in harmful environments. Therefore, it is usually chosen to be integrated with ZnO to enhance the gas-sensing ability of ZnO [2,16]. Recent works on gas-sensing performance of ZnO-NiO sheet-like composites demonstrate superior sensing responses to that of their individual counterparts [16]. The construction of ZnO-NiO heterogeneous p–n composite oxides will form an inner electric field in them. Such an interfacial potential barrier will affect the resistance variation of the p-n composite oxides on exposure to target gases and results in improved gas-sensing responses. The effect of p/n oxides content ratio in the composite system also plays a key that affects the resultant gas-sensing performance towards specific target gases [2]. Furthermore, the gas-sensing response is highly associated with the microstructure of oxides; the fabrication of unique microstructure with porosity and large specific surface area is beneficial to improve the sensitivity of sheet-like ZnO sensing material. Based on the aforementioned reasons, the construction of porous ZnO-NiO composite nanosheets with tuning NiO loading content is a promising study to develop 2D ZnO-NiO composite gas sensors with high performance. However, systematical investigation on microstructure-dependent gas-sensing performance of 2D porous ZnO-NiO composite system with variable NiO loading contents is still lacking. This is associated with the fact that most multiple chemical routes-synthesized 2D ZnO-NiO system hinders the precise control of microstructure evolution by changing the NiO crystallite loading content. The NiO coverage layer size-dependent microstructures and gas sensing responses are correlated herein. The ZnO-NiO composites with a suitable NiO crystallite loading content and a visible porous sheet structure demonstrate markedly improved gas-sensing performance towards ethanol vapor.

## 2. Materials and Methods

The ZnO nanosheets were grown on the F-doped tin oxide (FTO) substrates using a hydrothermal method. Zinc nitrate hexahydrate (Zn(NO_3_)_2_·6H_2_O; 4.47 g) together with urea ((NH_2_)_2_CO; 16.67 g), are dissolved in 100 mL deionized water. The hydrothermal reaction was performed at 90 °C for 4 h herein. After the deposition, template products identified as Zn_4_CO_3_(OH)_6_·H_2_O nanosheets were formed. These template nanosheets were further heated at 500 °C for 30 min in ambient air to be transformed into the porous ZnO nanosheets. Furthermore, porous ZnO-NiO composite nanosheets were fabricated by rf magnetron sputtering NiO thin films onto the surfaces of the porous ZnO nanosheet templates. The 2 inch sized NiO ceramic disc was used as a target. The NiO coverage films were sputter deposited in mixed Ar/O_2_ ambient with a ratio of 6:1 at 350 °C with a working pressure of 15 mTorr. The sputtering power of NiO target was fixed at 90 W. The porous ZnO-NiO composite nanosheets with two NiO coverage layer sizes were systemized by changing the deposition duration from 20 min to 40 min, which corresponded to the sample codes of ZnO-NiO-1 and ZnO-NiO-2, respectively.

Crystal structure of nanosheets was studied by X-ray diffraction (XRD; Bruker D2 PHASER, Bruker, Karlsruhe, Germany). The detailed microstructures of various porous nanosheets samples were investigated by scanning electron microscopy (SEM; Hitachi S-4800, Tokyo, Japan) and high-resolution transmission electron microscopy (HRTEM; JEOL JEM-2100F, Tokyo, Japan), respectively. The X-ray photoelectron spectroscopy (XPS; PHI 5000 VersaProbe, Chigasaki, Japan) was used to understand the compositions of the samples. The Pt electrodes were coated on the surface of the sensing materials. The various porous nanosheet-based sensors were placed in a homemade vacuum chamber, and different ethanol vapor concentrations (50, 100, 250, 500, and 750 ppm) were introduced into the test chamber, using synthetic air (21% oxygen) as the carrier gas. The mass flow controller (MFC) was used to control the flow rate of carrier gas during the gas-sensing tests. The operating temperature of gas-sensing measurements varied from 150 to 250 °C. All the gas-sensing tests are under a fixed applied voltage of 5 V. The detailed setup of the measurement system has been described elsewhere [17]. The gas sensing response of the n-type porous nanosheet-based sensors towards ethanol vapor is defined as the Ra/Rg. Ra is the sensor resistance in the absence of target gas and Rg is that in the target gas.

## 3. Results and Discussion

**Figure 1:**
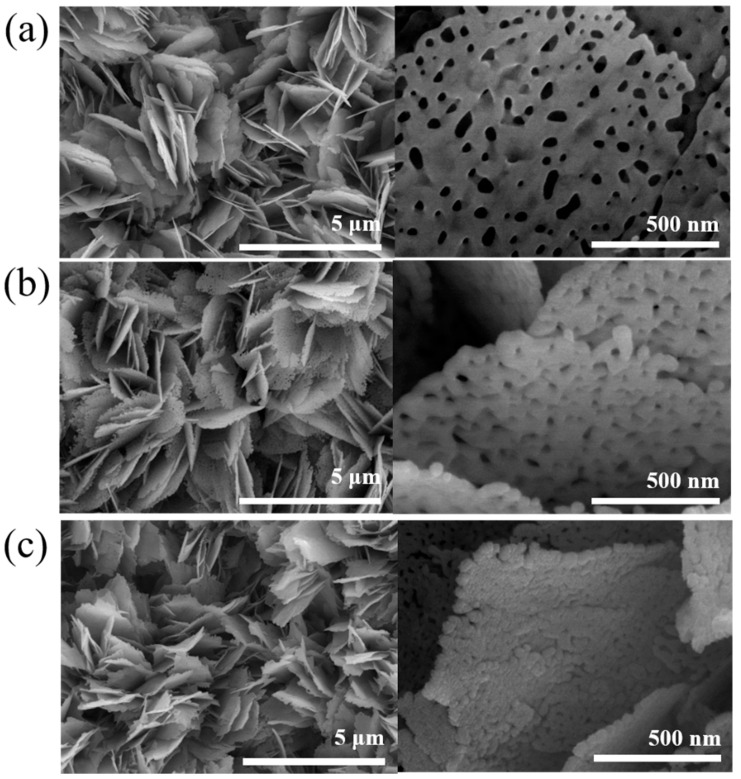
(**a**) SEM micrograph of the pristine porous ZnO nanosheets. The SEM micrographs of the porous ZnO-NiO composite nanosheets: (**b)** ZnO-NiO-1 and (**c**) ZnO-NiO-2.

Figure 1a shows the SEM micrograph of the pristine ZnO porous nanosheets. The nanosheets had a diameter ranging from 500–800 nm and covered on the substrate completely and uniformly. The nanosheets seem to grow radially in the vertical direction to the substrate surface and are standing upright on the substrate. The nanosheets had a round morphology with rugged periphery and the surface is smooth. The visible pores homogeneously distributed on the surfaces of the nanosheets. The thickness of the nanosheets was estimated to be approximately 20–25 nm. Figure 1b,c show the SEM micrographs of the porous ZnO nanosheets coated with NiO thin films with different sputtering durations. Coating NiO film on the ZnO porous nanosheet template changed sheet surface feature (Figure 1b,c) in comparison with the pristine ZnO porous nanosheets. The surface and sidewalls of the as-synthesized ZnO-NiO porous nanosheets became rough. The thickness of the ZnO porous nanosheets coarsened after sputtering coating the NiO thin film coverage layer. Furthermore, the pore size of the ZnO nanosheets was substantially shrunk with a prolonged NiO sputtering duration in Figure 1c. The whole surfaces of the ZnO porous nanosheets are uniformly and densely coated with a large amount of NiO crystals, revealing a surface feature with numerous aggregates on the sheets. The SEM analysis results displayed that the NiO crystals were successfully coated on the ZnO porous nanosheets and the surface feature of the ZnO sheets was changed because of the NiO crystal decoration.

**Figure 2:**
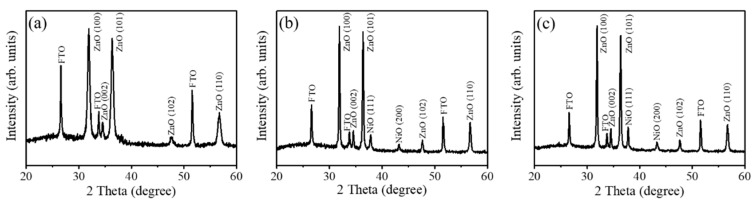
(**a**) XRD pattern of the pristine porous ZnO nanosheets. The XRD patterns of the porous ZnO-NiO composite nanosheets: (**b**) ZnO-NiO-1 and (**c**) ZnO-NiO-2.

Figure 2a shows the XRD pattern of the pristine ZnO porous nanosheets. Notably, in addition the Bragg reflections originated from the FTO substrate, other Bragg reflections in Figure 2a can be indexed to hexagonal wurtzite ZnO structure (JCPDS no. 01-079-0207). From Figure 2a, the intensity of the (100) and (101) Bragg reflections are substantially stronger than the (002) Bragg reflection, revealing the nonpolar plane crystal growth behavior for the sheet-like morphology of the wurtzite ZnO. A similar crustal growth feature has also been demonstrated in the electrodeposited Al-doped ZnO nanosheets [18]. The XRD patterns of the ZnO-NiO composite sheets prepared with various NiO coverage contents are shown in Figure 2b,c. In addition to the original ZnO Bragg reflections, two distinct Bragg reflections centered at approximately 37.2° and 43.3° are ascribed to the (111) and (200) planes of cubic NiO (JCPDS no. 00-011-0287). The distinct crystallographic feature of intense Bragg reflection of (111) is most exhibited in the sputtering deposited polycrystalline cubic NiO thin films [2]. No other impurity crystalline phases can be detected herein. Furthermore, the intensity of NiO Bragg reflections increased with an extended sputtering duration of the NiO film, revealing the thickened NiO coverage layer on the porous ZnO nanosheet templates as exhibited in Figure 2c. The XRD results herein demonstrate that crystalline ZnO–NiO dual phase composite nanosheets with were successfully synthesized via a sputtering-assisted decoration method in this study.

**Figure 3:**
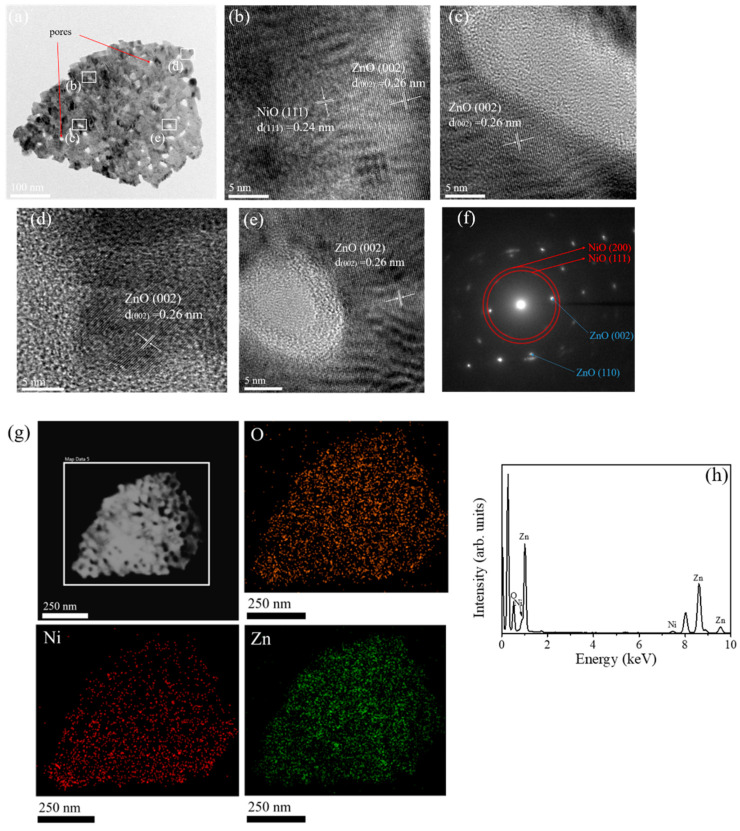
TEM analyses of a single ZnO-NiO-1 composite nanosheet: (**a**) Low-magnification TEM image. (**b**–**e**) The high-resolution (HR) images taken from various regions of the nanosheet. (**f**) SAED pattern. (**g**) EDS elemental mapping images collected by scanning the white square area and the corresponding elemental mapping images demonstrated that the Zn, Ni, and O were homogeneously distributed over the area of the whole sheet structure. (**h**) EDS spectra of Zn, Ni, and O taken from the ZnO-NiO-1 nanosheet.

The detailed microstructures of the ZnO-NiO porous-sheets were further characterized by TEM analysis. Figure 3a shows a low-magnification TEM image of a single ZnO-NiO-1composite sheet. The pores with a size ranged from 10–20 nm are randomly and homogeneously appeared in the sheet structure. The rugged peripheral morphology of the porous nanosheet was visible distinguished. The high-resolution (HR) images taken from the inner regions aside the pores and outer regions of the nanosheet are displayed in Figure 3b–e. The wrinkles with distinct gray scale fringe variation appeared around the pores, revealing the bending effect of the ultrathin oxide layer aside the pores. The lattice spacing of 0.26 nm and 0.24 nm in the sheet structure corresponded to the ZnO (002) and NiO (111) crystallographic planes, respectively. However, the regularity of lattice fringe arrangement in the most region of the nanosheet is not visibly distinguishable because of the overlapped stack of the ZnO and NiO oxide materials in the composite structure. The selected area electron diffraction (SAED) pattern in Figure 3f demonstrates the composite nanosheet consisted of the hexagonal ZnO and cubic NiO phases in it. The energy dispersive X-ray spectroscopy (EDS) elemental mapping images were further collected by scanning the white square area in Figure 3g. The corresponding elemental mapping images demonstrated that the Zn, Ni, and O were homogeneously distributed over the area of the whole sheet structure. The distribution width of Ni is slightly larger than that of Zn on the mapping images, further confirming that NiO crystals are sputtered on the surface of the ZnO porous nanosheet. The Ni content in the composite nanosheet was about 9.3 at% evaluated from the EDS spectrum (Figure 3h). Moreover, the result can prove that the synthesized nanocomposite is pure without any impurities.

**Figure 4:**
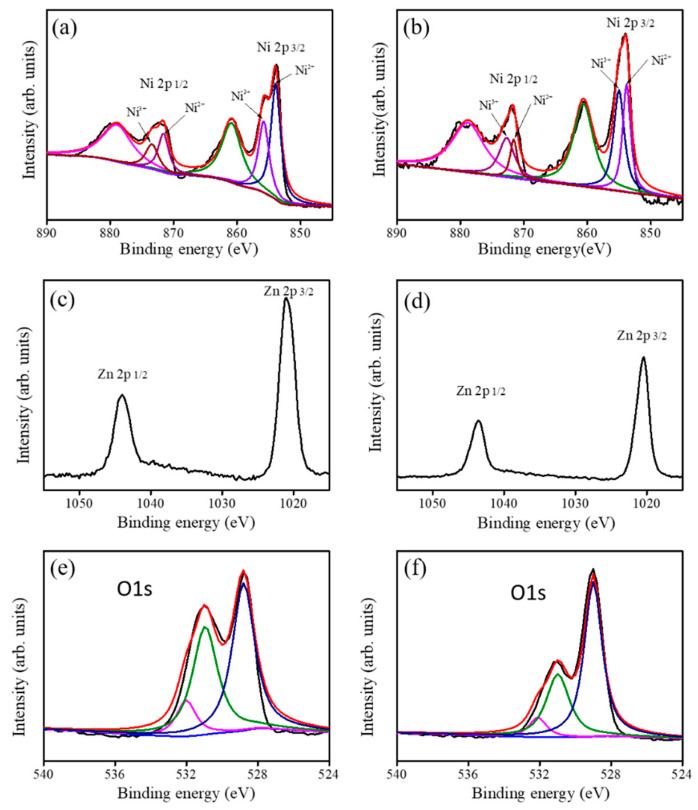
XPS analysis of the porous ZnO-NiO composite nanosheets: (**a**) Ni 2p narrow scan spectrum of the ZnO-NiO-1, (**b**) Ni 2p narrow scan spectrum of the ZnO-NiO-2, (**c**) Zn 2p narrow scan spectrum of the ZnO-NiO-1, (**d**) Zn 2p narrow scan spectrum of the ZnO-NiO-2, (**e**) O 1s narrow scan spectrum of the ZnO-NiO-1, (**f**) O 1s narrow scan spectrum of the ZnO-NiO-2.

The narrow scan XPS spectra of Ni 2p from the ZnO-NiO-1 and ZnO-NiO-2 porous nanosheets are displayed in Figure 4a,b, respectively. The Ni 2p spectra were further deconvoluted into six characteristic subpeaks. The two distinct binding subpeaks located at 853.4 eV and 855.3 eV corresponded to the Ni 2p_3/2_ feature, and the two binding subpeaks entered at approximately 870.7 eV and 872.7 eV corresponded to the Ni 2p_1/2_ feature. The remaining two binding subpeaks are ascribed to satellite features. The doublet subpeaks of 853.4 eV and 870.7 eV are related with Ni^2+^ component, and the subpeaks of 855.3 eV and 872.7 eV are ascribed to Ni^3+^ component in this study [19]. Notably, the full width at half maximum (FWHM) values of Ni^2+^ and Ni^3+^ components from Ni 2p_3/2_ feature are the same and are approximately 2.1 eV for the ZnO-NiO-1. Moreover, the FWHMs of Ni^2+^ and Ni^3+^ components are approximately 2.1 and 2.2 eV, respectively, for the ZnO-NiO-2. The existence of the Ni^3+^ binding status in the sputtering coated NiO thin film revealed the possible formation of the Ni vacancy in the NiO crystals during sputtering growth [20]. Comparatively, the relative intensity of Ni 2p spectrum of the ZnO-NiO-2 is higher than that of the ZnO-NiO-1 sample; this is attributed to an increased NiO crystal coverage amount with prolonged NiO sputtering duration for the ZnO-NiO-2. The narrow scan Zn 2p core-level spectra of the ZnO-NiO porous composite nanosheets are given in Figure 4c,d. The Zn 2p spectra showed two symmetric binding features, the peak centered at 1021.8 eV corresponds to the Zn 2p_3/2_ and the other one centered at 1044.8 eV is assigned to Zn 2p_1/2_. The spin-orbit splitting between Zn 2p_3/2_ and Zn 2p_1/2_ of various porous composite nanosheets is approximately 23 eV, indicating that zinc is in the Zn^2+^ binding state in the ZnO nanosheet templates [21,22]. Comparatively, the relative intensity of the Zn 2p spectrum in Figure 4d is weaker than that in Figure 4c; this is in agreement with the Ni 2p spectrum intensity variation for various ZnO-NiO porous composite nanosheets as exhibited in Figure 4a,b. The narrow scan O 1s XPS spectra of ZnO-NiO-1 and ZnO-NiO-2 are displayed in Figure 4e,f. The O 1s binding feature of various samples was further deconvoluted into three subpeaks. The subpeak centered at approximately 529.1 eV is assigned to the lattice oxygen binding to Ni and the subpeak located at 530.9 eV is attributed to the Zn–O binding status. The subpeak with the highest binding energy at 532.3 eV is associated with the surface adsorption oxygen species [23]. The Ni contents in the ZnO-NiO-1 and ZnO-NiO-2 are evaluated from the XPS analysis and are 9.1 at% and 14.9 at%, respectively.

**Figure 5:**
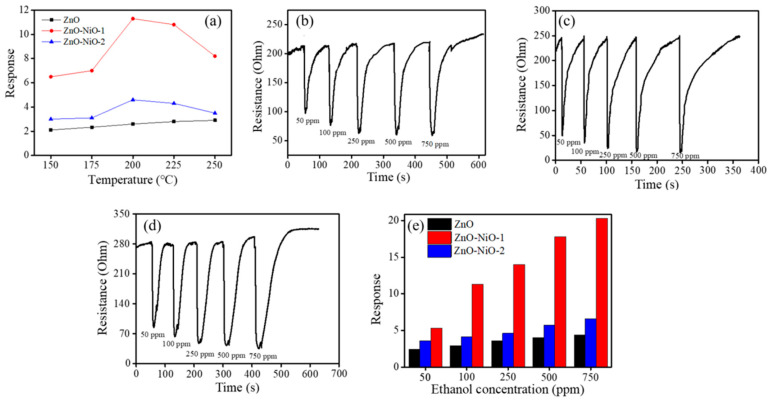
(**a**) Temperature-dependent 100 ppm ethanol vapor-sensing responses of various porous nanosheets. The corresponding dynamic electric resistance response-recovery curves of various porous nanosheets: (**b**) ZnO, (**c**) ZnO-NiO-1, and (**d**) ZnO-NiO-2. (**e**) Gas-sensing response values vs. ethanol vapor concentrations for the sensors made of various porous nanosheets.

As shown in Figure 5a, a summit appeared at 200 °C in the operating temperature dependent gas-sensing curves of the ZnO-NiO-1 and ZnO-NiO-2 sensors in the temperature range of 150–250 °C. At various operating temperatures, the response of the sensor based on the ZnO-NiO composites to ethanol vapor was higher than that of the sensor based on the pristine porous ZnO nanosheets. The observed temperature dependence gas-sensing behavior of the ZnO-NiO sensors was understood as follows: when the operating temperature is gradually increasing, the activation of the surface sensing is consequently accelerated, leading to an increased gas response. By contrast, when the operating temperature is above the optimal value, the ethanol vapor molecule desorption rate is faster than its adsorption rate on the ZnO-NiO surfaces. The chemisorbed oxygen ions on the surfaces of the porous ZnO-NiO nanosheets do not have sufficient reaction time to react with the ethanol vapor molecules and to release the charge carrier back to the oxides owing to a higher surface activation, hence resulting in a decrease in ethanol vapor sensing response [24]. Notably, no clear summit appeared in the gas-sensing response vs. operating temperature curve for the pristine porous ZnO nanosheets herein. The gas-sensing response of the porous ZnO nanosheets increased with an increased operating temperature in the temperature range of 150–250 °C, revealing the optimal operating temperature of the porous ZnO nanosheets is above 250 °C in this study. A similar reduced optimal operating temperature in the p-n oxide composite systems in comparison with that of the constituent counterparts associated with formation of a p-n junction has been demonstrated in the ZnO-ZnFe_2_O_4_ and PdO-ZnO composites [25,26,27]. The further ethanol vapor concentration dependent gas-sensing tests of the as-synthesized porous nanosheet-based sensors are maintained at 200 °C in this study. Figure 5b–d show the dynamic resistance response–recovery curves of the ZnO, ZnO-NiO-1, and ZnO-NiO-2 sensors upon exposure to different ethanol vapor concentrations at 200 °C. All the sensors displayed well cyclic ethanol vapor concentration-dependent sensing ability. A typical n-type semiconductor sensing behavior is observed for all sensors when exposed to ethanol vapor. The sensor resistance decreased on exposure to ethanol vapor. It is known that the ZnO and NiO have, respectively, n- and p-type semiconducting natures. The n-type gas-sensing behavior of the ZnO-NiO porous sheets exposed to the ethanol vapor herein demonstrated that the NiO crystallites are not fully covered the whole surfaces of the porous ZnO nanosheet template. This is similar to the gas-sensing behavior in the TiO_2_–Ag_2_O composite nanorods with p-type Ag_2_O crystallite decoration not fully covered the surfaces of the TiO_2_ nanorod templates [3]. The exposure of the surfaces of the n-type templates in the composite structure might affect the electric properties of the p-n composite structure during reducing gas-sensing tests. Furthermore, the ethanol vapor sensing responses of various sensors herein increased with the ethanol vapor concentration, revealing that an increased ethanol molecules interacted with the surface absorbed oxygen species. Notably, the sensors made from the ZnO-NiO composite porous nanosheets showed a relatively wide resistance variation range towards ethanol vapor at the given test condition in comparison with that of the pristine porous ZnO nanosheets. This demonstrates the improved ethanol vapor sensing ability in the porous ZnO nanosheets via sputtering decoration of the NiO crystallites. Figure 5e illustrates the ethanol vapor sensing responses of the sensors made of various porous ZnO-NiO composite nanosheets are superior to the sensor made of the pristine porous ZnO nanosheets. This is attributed to the existence of the ZnO/NiO heterojunction potential barrier in the composite nanosheets. Similarity, the p-type Mn_3_O_4_/n-type SnO_2_ heterogeneous system exhibits improved H_2_ gas-sensing performance, superior to bare Mn_3_O_4_ [28]. The gas sensor based on the Co_3_O_4_-ZnO p-n composite has higher sensitivity to triethylamine compared with pure ZnO sensor; the optimized triethylamine sensing performances can be ascribed to the p-n heterojunction effect between Co_3_O_4_ and ZnO [29]. The PdO/ZnO p–n heterojunction in the PdO/ZnO composite nanostructures improves acetaldehyde gas-sensing performance of the pristine ZnO nanostructures [30]. An extra potential barrier at the ZnO/ZnCr_2_O_4_ interface is shown to be an important factor for the gas-sensing performance towards reducing gases [17]. Comparatively, the porous ZnO-NiO-1 composite nanosheet-based sensor showed a higher gas detecting ability towards ethanol vapor than did ZnO-NiO-2 sensor under the same gas-sensing test conditions. The gas-sensing responses of the ZnO-NiO-1 sensor ranged from 5.3 to 20.4 upon exposure to 50–750 ppm ethanol vapor, respectively. Further prolonging NiO sputtering duration (ZnO-NiO-2), the ethanol vapor-sensing responses slightly decreased in comparison with those of the ZnO-NiO-1 under the same gas-sensing test conditions (Figure 5e). The inferior ethanol vapor-sensing responses of the ZnO-NiO-2 can be associated with several factors. The one factor is that an increased NiO loading content markedly decreased the porosity of the composite nanosheets because a thick coverage of the NiO crystallites blocked the pores of the porous ZnO nanosheet template as revealed in earlier SEM images. Furthermore, the optimal content ratio between the constituent oxide compounds in oxide heterogeneous systems is a key role in increasing the sensing ability toward target gases [31]. The thick p-type oxide coverage layer might reduce the p-n junction thickness variation sensitivity in the p-n oxide heterostructures on exposure before and after to target gases. For example, in one-dimensional TiO_2_-Ag_2_O p-n heterogeneous system, too thick Ag_2_O coverage layer on TiO_2_ nanorods further decreases the gas-sensing performance of the TiO_2_-Ag_2_O nanorods [3]. Based on the aforementioned possible reasons, the ZnO-NiO-2 sensor showed inferior ethanol vapor sensing performance than that of the ZnO-NiO-1 sensor. The response time of the sensor is defined as the duration when the resistance reaches 90% of its maximum response. Meanwhile, the recovery time is defined as the duration when it returns to 90% of the original value upon removal of ethanol vapor. The response and recovery times are respectively in the ranges of 12–23 s and 101–156 s for the ZnO sensor exposed to 50–750 ppm ethanol vapor. Notably, the ZnO-NiO-1 sensor not only exhibited a substantially higher ethanol vapor sensing response but also displayed faster response and recovery speeds. The response and recovery times of the ZnO-NiO-1 sensor are in the ranges of 4–13 s and 78–121 s exposed to 50–750 ppm ethanol vapor, respectively. These results revealed that the response and recovery speeds of the pristine ZnO porous sheets can be improved by the formation of the ZnO–NiO composite porous sheets with a suitable loading of NiO crystallites, and this is similar to the results of CuO–ZnO heterojunctions [32].

**Figure 6:**
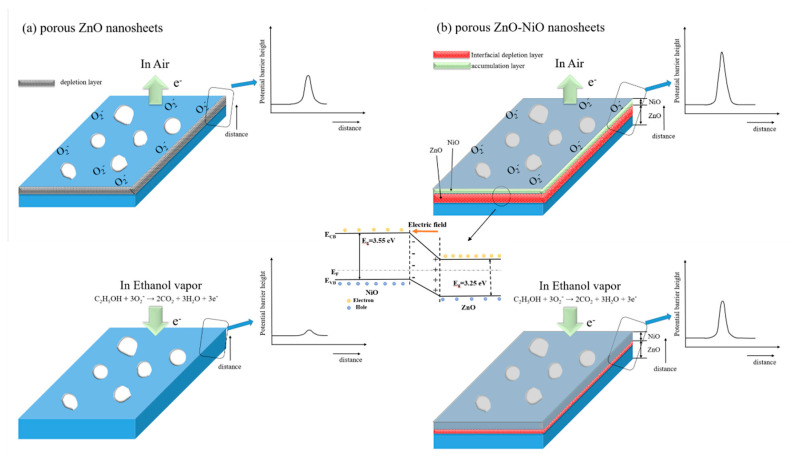
Schematics of possible ethanol vapor-sensing mechanisms of the (**a**) porous ZnO nanosheet and (**b**) porous ZnO-NiO composite nanosheet.

The gas-sensing mechanism of metal-oxide semiconductor can be understood by the bulk resistance change associated with the interaction between adsorbed oxygen species and target gas molecules on the oxide surface [3,4]. As a representative n-type semiconductor oxide, the ethanol vapor sensing behavior of the porous ZnO nanosheet is displayed in Figure 6a. When porous ZnO nanosheets based gas sensor is exposure to ambient air at 200 °C; oxygen molecules adsorbed on the surface would be ionized into oxygen species in the form of O_2_^-^ through capturing negative electrons from the conduction band of ZnO [33]. Hence, a surface depletion layer is generated, consequently causing an increase in sensor bulk resistance because of formation surface potential barrier height. While the reductive ethanol vapor is introduced, the ethanol vapor molecules will react with the adsorbed oxygen species, especially the porous structural feature enhanced the flow characteristics of ethanol vapor molecules on the oxide surface of porous ZnO nanosheets; this might accelerate the ethanol vapor molecules to interact with the surface adsorbed oxygen ions of the ZnO. The snatched electrons will be liberated back to the conduction band of the ZnO according to Equation (1), thus decreasing the depletion layer width and then the bulk sensor resistance:

C_2_H_5_OH(vapor) + 3O_2_^−^ (ads) → 2CO_2_ + 3H_2_O + 3e^−^ (1)

By contrast, in the case of the porous ZnO-NiO composites, two types of potential barrier heights might affect the bulk resistance variation of the sensor. Firstly, the adsorbed oxygen ions induced the extraction of free electrons in the surface region of the NiO decoration layer; this might increase the surface hole concentration in the NiO crystallites. The contact of the surface hole accumulation NiO crystallite layer with the porous ZnO nanosheet template engendered the formation of p-n heterojunction with a marked depletion size at the interface between the n-type ZnO and p-type NiO. This can be understood with the fact that n-type ZnO and p-type NiO have different work functions (ZnO = 4.4 eV, NiO = 5.2 eV) [34]. Moreover, the bandgap values of the porous ZnO sheet template and sputtering NiO film are 3.25 eV and 3.55 eV, respectively, according to our previous works [20,35]. An internal electrical field at the ZnO/NiO interface was formed and induced the band bending at the interface region (Figure 6b). This process will lead to both high barrier height and contribute to a high initial resistance during the ionization of oxygen in comparison with the pristine ZnO (Figure 6a). After being exposed to ethanol vapor, the released electrons back to partial regions of the root of the ZnO nanosheet template in which without the well coverage of NiO. Simultaneously, the holes in p-type NiO coverage region of the top regions of ZnO nanosheet template combined with the released electrons, causing a drop in hole concentration; this process weakened the diffusion of carries and evidently shrinks the potential barrier height of ZnO/NiO interfacial depletion layer. The lowing of ZnO surface potential barrier height together with the p-n interfacial potential barrier height further sharply decreased the total bulk sensor resistance (Figure 6b) [36]. On the basis of above analysis, the p-n heterojunction in the composite system visibly increased total bulk resistance variation degree of the porous ZnO-NiO composite nanosheets in comparison with that of the pristine porous ZnO nanosheets during gas-sensing tests. Therefore, an enhanced ethanol vapor detecting ability was observed for the porous ZnO nanosheets via sputtering decorated NiO crystallites on them.

**Figure 7:**
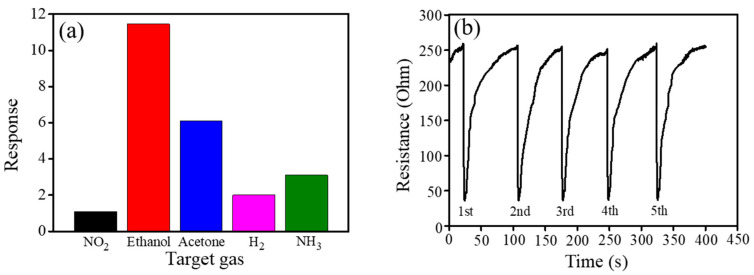
(**a**) The cross gas-sensing sensitivity of the ZnO-NiO-1 sensor towards various target gases. (**b**) Cyclic ethanol vapor-sensing curves for the ZnO-NiO-1 sensor towards 100 ppm ethanol vapor.

Table 1 shows comparative ethanol vapor sensing performance of various p-n composites comprised with NiO synthesized via various routes [37,38,39]. The porous ZnO-NiO-1 composite nanosheets herein demonstrate higher sensing response at a lower optimal operating temperature towards lower ethanol vapor concentration in comparison with other works. The cross sensitivity of the ZnO-NiO-1 sensor was further investigated on exposure to various reducing gases (NH_3_, C_2_H_5_OH, H_2_, and C_3_H_6_OH: 100 ppm) and 15 ppm oxidizing gas (NO_2_), and the results were shown in Figure 7a. Comparatively, the ZnO-NiO-1 sensor was more inactive to detect NH_3_, H_2_, and NO_2_ harmful gases at the given test conditions. Furthermore, it has been shown that ethanol and acetone are similar in chemical nature; the sensing discrimination between ethanol and acetone is not easy for the most metal oxides [40]. The ratio between gas-sensing response to alcohol and that to acetone was used as a norm to assess the sensor selectivity. In this work, that ratio is approximately 1.95, revealing an almost two folds higher response of the ZnO-NiO-1 sensor to detect ethanol vapor than that to detect acetone vapor. This result might indicate the decent ethanol vapor selectivity in the test environment containing acetone vapor in it. Moreover, as illustrated in Figure 7b, the ZnO-NiO-1 sensor had excellent sensing repeatability as well as recovery performances towards 100 ppm ethanol vapor at 200 °C for 5 cycles. The results herein revealed that decoration of porous ZnO nanosheets with a suitable content of NiO crystallites via a sputtering-assisted method is a promising route to fabricate 2D porous ZnO-NiO composites for gas sensor applications.

## 4. Conclusions

In summary, the effects of sputtering deposited p-type NiO coverage layer on the gas-sensing functionality of the porous ZnO-NiO composite nanosheets are disclosed herein. The porous ZnO-NiO composite nanosheets were synthesized by coating crystalline NiO films on the porous ZnO nanosheet templates. The NiO sputtering duration was changed to adjust the NiO coverage content on the porous ZnO nanosheet templates. The porous composite nanosheets with a 20 min NiO sputtering duration (ZnO–NiO-1) exhibited superior gas sensing properties than that of other control samples. The mesopores on each porous ZnO-NiO composite nanosheet provided plenty of active sites for surface chemical reactions and efficient target gas molecular diffusion channels to increase the sensing efficiency. The p–n heterojunction in the porous ZnO-NiO composite nanosheets increased the total potential barrier number as compared to the pristine ZnO, and this is beneficial to gas-sensing response towards ethanol vapor.

## Figures and Tables

**Table 1:** Comparative ethanol vapor sensing performance of various p-n composites comprised with NiO synthesized via various synthesis routes [37,38,39].

Material	Synthesis Method	Morphology	Operating Temperature/Ethanol Concentration	Response (Ra/Rg)	Response Time(s)	Recovery Time(s)
**NiO-ZnO**	Coelectrospinning	nanotubes	215 °C/200 ppm	<5	N/A	N/A
TiO_2_-NiO	Hydrothermal method	nanorods	400 °C/200 ppm	6.4	N/A	N/A
WO_3_-NiO	Coelectrospinning	nanofibers	375 °C/100 ppm	8	N/A	N/A
**ZnO-NiO** **(this work)**	Hydrothermal method/Cosputtering	porous nanosheets	200 °C/100 ppm	11.3	4	78
